# Regulating the Electronic Structure of Freestanding Graphene on SiC by Ge/Sn Intercalation: A Theoretical Study

**DOI:** 10.3390/molecules27249004

**Published:** 2022-12-17

**Authors:** Xingyun Luo, Guojun Liang, Yanlu Li, Fapeng Yu, Xian Zhao

**Affiliations:** 1 State Key Lab of Crystal Materials, Institute of Crystal Materials, Shandong University, Jinan 250100, China; 2 Center for Optics Research and Engineering of Shandong University, Shandong University, Qingdao 266237, China

**Keywords:** graphene, intercalation, SiC substrate, electronic structure, density functional theory

## Abstract

The intrinsic n-type of epitaxial graphene on SiC substrate limits its applications in microelectronic devices, and it is thus vital to modulate and achieve p-type and charge-neutral graphene. The main groups of metal intercalations, such as Ge and Sn, are found to be excellent candidates to achieve this goal based on the first-principle calculation results. They can modulate the conduction type of graphene via intercalation coverages and bring out interesting magnetic properties to the entire intercalation structures without inducing magnetism to graphene, which is superior to the transition metal intercalations, such as Fe and Mn. It is found that the Ge intercalation leads to ambipolar doping of graphene, and the p-type graphene can only be obtained when forming the Ge adatom between Ge layer and graphene. Charge-neutral graphene can be achieved under high Sn intercalation coverage (7/8 bilayer) owing to the significantly increased distance between graphene and deformed Sn intercalation. These findings would open up an avenue for developing novel graphene-based spintronic and electric devices on SiC substrate.

## 1. Introduction

The linear dispersion of π-bands endows graphene with novel physical properties and vast potential for applications in electric, optoelectronic, and photo-electrochemical fields [1,2,3,4,5]. One of the most effective ways to achieve linear π-bands of graphene is the epitaxial growth of quasi-freestanding graphene on a Si-terminated SiC substrate [6,7]. However, a buffer carbon layer with a graphene-like hexagonal honeycomb arrangement would inevitably be formed between the SiC substrate and graphene. The interfacial states of the buffer layer can lead to a charge transfer from the underlying (6√3 × 6√3) R30° SiC (0001) surface to graphene, resulting in a strongly n-doped graphene [1,2,3,8,9,10,11]. Modulating the p-type and charge-neutral graphene is still challenging. Furthermore, because the Fermi level shifts upward from delocalized π-bands into valance bands, the carrier mobility of graphene is considerably reduced [12].

Generally, the intercalation between the buffer layer and SiC substrate is beneficial for turning the buffer carbon layer into freestanding graphene by saturating the Si dangling bonds. Graphene may be doped with electrons or holes depending on the type and properties of intercalations. The most widely used H intercalation [13,14,15,16,17] results in almost electrically neutral graphene (weak p-type, Fermi level ~0.11 eV below the Dirac point), while an intercalation with other gases [18,19,20,21,22,23], such as O and F, with strong oxidation ability, produces p-doped graphene. At the same time, various metal intercalations [3,24,25,26,27,28,29,30,31,32] have been studied, and they provide stability with respect to the gas intercalations and simple preparation process with respect to the compound intercalations, such as FeSi and B_x_C_y_, as reported in our previous work [33,34]. However, most metal intercalations are not very effective in regulating the stable p-type and charge-neutral graphene. For example, alkali metal intercalations are very reactive and could easily release their valence electrons to produce n-type graphene, or break the Dirac-point of graphene [3,24,35,36,37]—the band gap of graphene increased up to 0.32 eV with increasing K intercalation coverage [3], and Na intercalation led to a band gap of 0.29 eV for graphene [37]. Transition metal (TM) intercalation, such as Mn, led to a Dirac half-metal character of graphene caused by the transition-metal d characteristics [28,31]; Fe intercalation induced charge asymmetry and multiple spin-polarized p bands in the electronic structure of graphene/Fe/SiC [32]. Overall, due to the extremely strong metallic properties or the partly-filled d-orbitals, alkali metal and TM intercalations can easily cause the polarization doping of graphene, making it challenging to regulate charge-neutral and the ambipolar doping of graphene.

To achieve the stable p-type and charge-neutral graphene, the intercalation materials need to meet the following basic screening conditions. Firstly, the intercalated atomic orbitals should have strong electron providing and holding capacity at the same time. Secondly, the intercalation layer could stably exist between the SiC substrate and graphene. Finally, the intercalation could only modulate the electronic structure without introducing any magnetism to graphene. The intercalation of the main group metals without d orbitals may overcome the negative influence on the insurmountable electron doping and magnetic interactions of graphene. The Ge [9,38,39,40,41,42] and Sn [43,44,45] intercalations as well as the uniform Sn_1-x_Ge_x_ alloy intercalations with different Sn:Ge ratios [46] could be successfully synthesized between the buffer layer and SiC substrates by chemical vapor deposition and template methods. The Ge intercalation was found to induce ambipolar doping of graphene, and the transition from p-type to n-type originated from a strong electron correlation of the Ge atoms [38]. Furthermore, the charge-neutral freestanding graphene was prepared by Sn intercalation since the conductive electrons of the Sn layer completely compensate for the spontaneous polarization charge of the SiC substrate [43]. Although the experiments suggest the successfully modulation of the electronic structure of free-standing graphene by Ge and Sn intercalations, there are still a few unanswered questions: (1) How can the conduction type (n-type, p-type, and charge-neutral) of graphene be modulated, and what is the modulating mechanism? (2) What are the effects of the coverage and location of these metal intercalations? (3) Would these intercalations introduce magnetism into graphene? (4) How stable are the intercalation structures under the graphene growth temperature?

To answer these questions, we performed first-principles calculations to investigate the structures and electronic structure of the Ge, Sn, and Sn_1-x_Ge_x_ intercalated systems. The effect of the coverage of intercalations and the location of the intercalated atoms on the electronic structure of graphene has been highlighted. The calculation results verified that the p-type and charge-neutral graphene could be achieved by high-coverage Ge and Sn intercalations, which is attributed to the charge transfer mechanism. Both the Ge and Sn intercalations could effectively inhibit the induced magnetism in graphene, which was different from the cases of TM intercalations [28,29,30,31,32]. These results provided theoretical evidence and guidance for modulating the electron doping character of epitaxial graphene on SiC substrate, thus promoting the practical application of graphene in microelectronic devices.

## 2. Results and Discussion

### 2.1. Electronic Structure Modulation by Ge Intercalations

**Figure 1:**
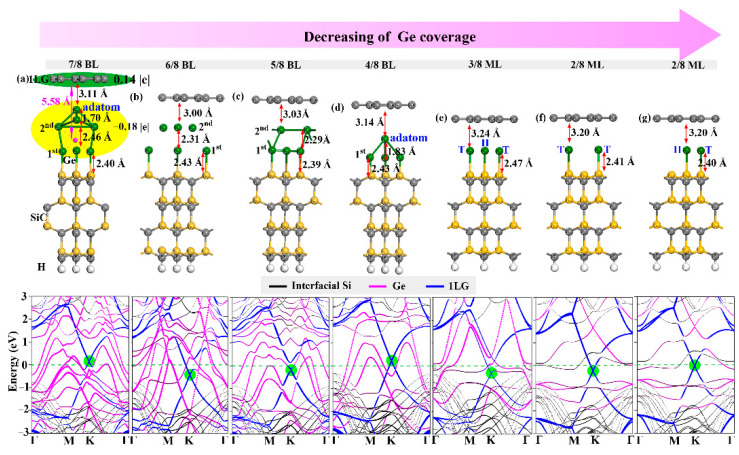
(**a**–**g**) Structures and band structures of 1LG/Ge/SiC with different Ge locations and coverages. In (**a**), Bader charges (e) carried by 1LG (green area) and Ge intercalation (yellow area) have been labeled. The rose red ball represents the mass center of the intercalation. In the band structures, the pink, blue, and black lines represent the contribution of Ge intercalation, 1LG, and interfacial Si of SiC substrate, respectively. The green circles show the graphene Dirac point.

The band structures of (2 × 2)Gr − (√3 × √3)SiC are compared to the (2 × 4)Gr − (√3 × 2√3)SiC structure to evaluate the effect of the computational cell size on the electronic structure of graphene (Figure 1c,d). It is found that the dominant electronic contributions near the Fermi level and the relative position of the Dirac point of graphene to the Fermi level remain unchanged (0.45 eV below the Fermi energy) for the structures calculated using different computational cells. The shapes of the bands changed slightly due to the increased band density of the (2 × 4)Gr − (√3 × 2√3)SiC structure. This alteration has little influence on the main conclusion, and the effect of the computational cell size on the electronic structure can thus be neglected.

The Ge intercalations can decouple the interactions between the 0LG and SiC substrates, as shown in Supplementary Materials Figure S2b. The formation energies and intercalation energies of the graphene/Ge/SiC system depicted in the Supplementary Materials are shown in Table 1. According to the formation energies, it was found that the 3/8 ML coverage for the Ge intercalations is the most favorable, and increasing or decreasing the coverages lead to the structure energetically less preferable. This indicates that the intercalation atoms prefer to saturate all Si dangling bonds. It is noted that the stability of Ge intercalated structures with 7/8 BL coverage, which corresponds to BL intercalation with an extra adatom, is significantly increased. The essential modulation mechanism needs to be further discussed carefully. Meanwhile, the very low coverages of metal intercalations (lower than 1/8 ML and 1/16 ML for Ge intercalations respectively) are quite unstable (negative values of *E*_f_), and we will not discuss these cases anymore. The intercalation energies indicated that all the intercalation structures are stable, and the relative stability of the intercalation structures depends on the coverage—high coverages show better thermodynamic stability. Then, their thermal stabilities were tested using AIMD simulations. It was reported that graphene was experimentally synthesized by the thermal decomposition of SiC at 900 °C, and Ge (Sn) intercalation was generated at 600–800 °C [46]. The AIMD simulations were thus conducted at 300, 900, and 1200 K, lasting 10 ps, and the results for 1LG/Ge/SiC with 3/8 ML and 6/8 BL coverages are chosen and shown in Supplementary Materials Figure S3(a,b) For both structures, the energies oscillated within small ranges at investigated temperatures, verifying the high thermodynamic stabilities under preparation and application temperatures. See Table 1.

**Figure 2:**
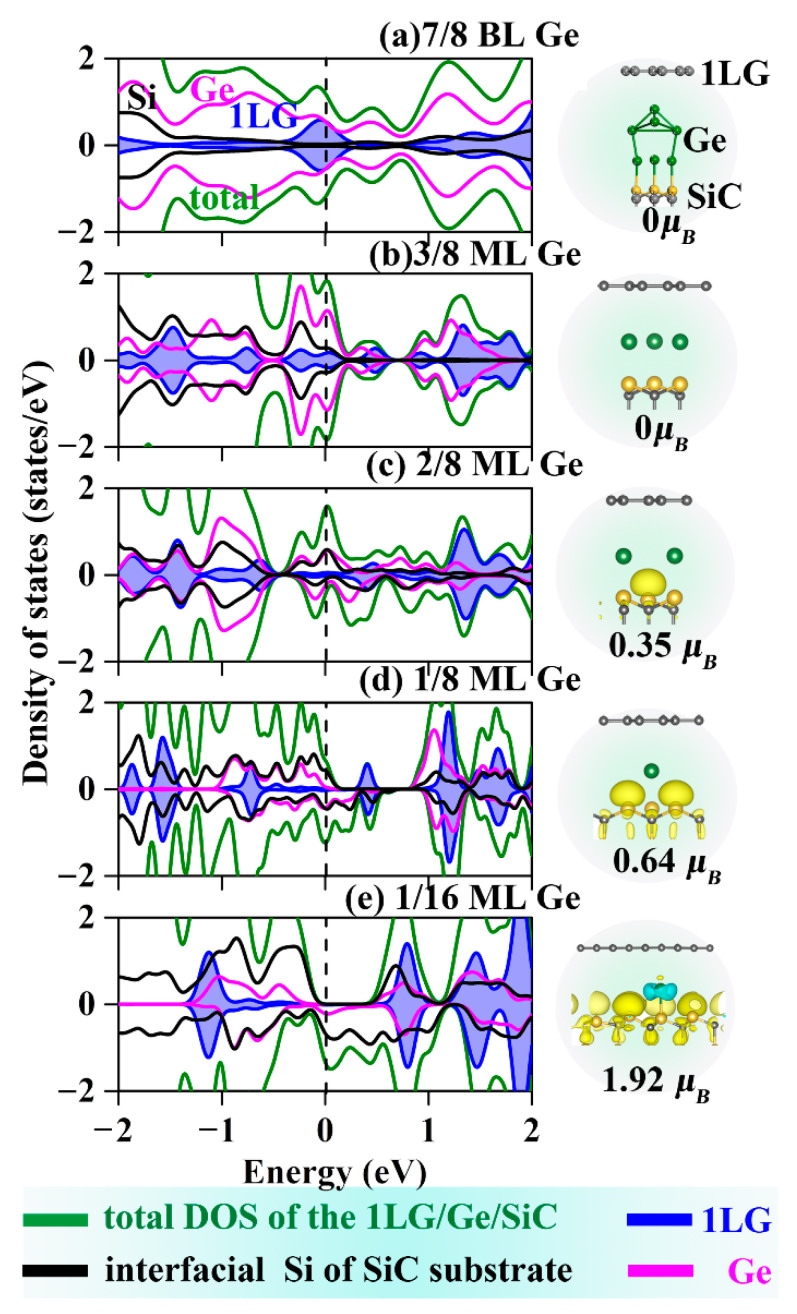
DOSs and spin densities for (**a**–**e**) 1LG/Ge/SiC with different coverages. Light blue and yellow isosurfaces represent positive and negative spin densities (±0.02 e/Å3), respectively. The green lines represent the total DOS of 1LG/Ge/SiC. The black, pink, and blue lines correspond to the PDOS of interfacial Si of SiC substrate, Ge, and 1LG, respectively.

Here, the effect of the intercalation coverage and atom location on the electronic structure modulation of graphene has been explored. The experiments showed that some Ge atoms deintercalated from the system with increasing temperature, resulting in a decrease in the Ge coverage gradually [38]. Angle-resolved photoemission spectroscopy (ARPES) can be used to accurately track the number of intercalated atoms to study the effect of intercalation coverage on the electronic structure of graphene [25,47], thus we considered Ge intercalations with decreasing coverages: 7/8 BL, 6/8 BL, 5/8 BL, 4/8 BL, 3/8 ML, 2/8 ML, 1/8 ML, and 1/16 ML. For the case of 2/8 ML, 1/8 ML, and 1/16 ML coverages, we studied the different cases of Ge atoms located at the T or H position. The optimized configurations and corresponding band structures of unfavorable 1/8 ML and 1/16ML coverages are shown in Supplementary Materials Figure S4(a,b,c,d) and all the others are presented in Figure 2. In 1LG/Ge/SiC structure, Ge atoms formed Ge-Si chemical bonds (bond length ~2.4 Å) on the top of the SiC substrate and weak interaction with 1LG. Such weak interaction is reflected by the flat structure of graphene and the distance of 3.0–3.2 Å between Ge and 1LG, comparable to that of 3.35 Å between the graphene layers [48]. The decoupling of 0LG from the SiC substrate can be confirmed by such structural characters and the typical Dirac point of graphene in the band structure of 1LG/Ge/SiC (Figure 1). Because Ge has much more metallic properties than C, the electrons prefer to transfer from the Ge intercalation to graphene, leading to the graphene Dirac point below the Fermi level, and the Fermi level passing through the Ge dangling bond states.

The Ge-intercalated structures show a coverage-dependent electronic ambipolar doping of graphene. As shown in Figure 1 and Supplementary Materials Table S1, a change in the Ge intercalation coverage from high to low induced a reduction in Fermi level and a transition from p-type to n-type graphene, which is consistent with the experimental reports [9,38]. In most cases, the graphene exhibits n-type owing to the more metallic character of Ge relative to C, which makes electron transfer from the Ge layer to graphene. With decreasing the Ge coverage from 6/8 BL to 1/16 ML, the electron doping of graphene is weakened, and graphene thus switched from n-type to weak p-type at 1/16 ML coverage. However, the 1/16 ML Ge intercalation was energetically unfavorable, implying that modulating the p-type graphene by extreme low coverage of Ge intercalation is impossible. This is because when more Ge atoms are missing, more Si electronic states from the Si dangling bonds contribute to the electron transfer and the Dirac point of graphene (Supplementary Materials Figure S4c,d).

When the Ge coverages are as high as 7/8 and 4/8 BL, the graphene becomes p-type with the Dirac point at 0.18 eV above the Fermi level (Figure 1a,d). The transition from n-type to p-type graphene is attributed to the stabilization of the Ge adatom between graphene and the Ge layers. We take the 7/8 BL coverage as an example to illustrate the origin of p-type graphene. As shown in Figure 2a, the Ge adatom is riveted by three Ge atoms in the second Ge layer, and the bond lengths between the Ge adatom (Ge_1_) and other Ge atoms (Ge_2_, Ge_3_, and Ge_4_) in the second Ge layer were ~0.8 Å smaller than those between the Ge atoms in the same layer (Table 2). In order to analyze the interaction between Ge atoms, the projected crystal orbital Hamilton population (COHP) was performed with the LOBSTER program [49] and VASP outputs. The COHP values between atoms can be obtain by partitioning the wave function, pregenerated from a self-consistent DFT calculation, into bonding, nonbonding, and antibonding contributions. It is defined as Equation (1).

(1) $$-{-\mathrm{COHP}}_{\mathrm{ij}}\left(E\right){=H}_{\mathrm{ij}}\sum_{n}c_{i}^{n}c_{j}^{*n}{\delta (E-E}_{n})$$

where H_ij_ is the Hamilton matrix element between atomic orbitals ∅_i_ and ∅_j_, and c_i_^n^ are the coefficients associated with ∅_i_ in a molecular orbital. The integrated COHP (ICOHP) calculated using the following Equation (2).

(2) $$\mathrm{ICOHP}\left(\varepsilon_{f}\right)=\int_{\infty }^{\varepsilon_{f}}\mathrm{COHP}(E)\mathrm{dE}$$

It is usually treated as a descriptor of the bond strength in compounds, and the negative value of ICOHP indicates strong interaction between atoms. The calculated ICOHP values of Ge_1_-Ge_2/3/4_ are an order of magnitude smaller than the others, demonstrating that Ge_1_ and Ge_2/3/4_ have strong chemical bonding interactions. Such strong chemical bonding of the Ge adatom to the Ge layer induces the charge transfer from graphene to the Ge intercalation. Bader charge calculation results show that the graphene loses 0.14 |e|, while the Ge intercalation gains 0.18 |e|, demonstrating the formation of p-type graphene in the presence of the Ge adatom. Meanwhile, the presence of the Ge adatom for the 4/8 and 7/8 BL coverages results in a significant change in the distance between the layers. Specifically, the distances between the Ge layers are largely decreased by 0.3–0.6 Å, and the distances between graphene and the Ge layer are slightly increased by 0.11–0.14 Å.

For the low Ge coverages (2/8, 1/8, and 1/16 ML), the effect of intercalation atom location on the band structures is also investigated. We found that the atomic location only slightly influences the position of the Dirac point, and the electronic states near the Fermi level as well as the conduction type of graphene remain almost the same. This observation is consistent with the experimental conclusion that the graphene doping type is not sensitive to the order of the intercalation layer structure [35]. Since the formation of such low Ge coverages becomes relatively difficult, we can conclude that the doping properties of graphene is dominantly modulated by the coverage of Ge intercalation instead of the location.

Since Ge has metal characteristics that may introduce magnetism into the graphene analogously to the Fe intercalation, the spin DOS and the electron density of Ge-intercalated structures were calculated and showed in Figure 2. It is seen that the ML coverage is the cut-off point for whether or not magnetism will be introduced into the Ge intercalated systems—coverages higher than ML do not induce magnetism to the entire structure, while coverages lower than ML, namely ML intercalation with Ge vacancies, the symmetry of the spin-up and spin-down electronic states has been broken, and the magnetism of the entire intercalation structures has thus been increased. The magnetic moments of the systems with the same coverage are almost the same, as shown in Table S1. This is because the exchange interaction between the electrons in the metal intercalations and the unsaturated dangling bonds of SiC leads to the polarization of the electronic states at the Fermi level, thus inducing the magnetism of the entire system. The number of Si dangling bonds increases with decreasing the intercalation coverage, and the magnetism of the entire system is thus increased. The asymmetric DOS spectra of the two spin components in Figure 2e confirmed the maximum magnetic behavior of the Ge intercalated structures with magnetic moments of 1.92 μ_B_. However, the magnetism has not been extended to graphene owing to the complete graphene π-bonds and little interaction with metal intercalations. When the Ge coverage reduces to 1/16 ML, the Ge-intercalated structure becomes half-metal, which arises from the electronic and magnetic couplings of the Ge layer with the SiC surface rather than the graphene. It indicates that such a structure may fabricate spin batteries and ideal magnetic tunnel junctions for spintronic applications.

### 2.2. Electronic Structure Modulation by Sn Intercalations

Similarly, the Sn intercalation can also transform 0LG into free-state graphene, as shown in Supplementary Materials Figure S3c. The Sn intercalations also completely saturate the Si dangling bonds by forming strong Sn–Si covalent bonds, whereas maintain a Sn dangling bond perpendicular to the graphene rather than form Sn-C bonds. This observation is sufficient to illustrate that the energy loss in the sp^2^ planarity of the graphene is higher than the energy gain from forming the covalent Sn-C bonds. Meanwhile, the E_f_ and E_I_ of Sn intercalation system are also used to reflect the possible and preferable coverage ranges of the intercalations in terms of energy, as shown in Table 3. The conclusions are consistent with those of Ge intercalation system. Namely, the 3/8 ML coverage for the Sn intercalations is the most favorable, and increasing or decreasing the coverages lead to the structure energetically less preferable. In addition, the thermodynamic stability for 1LG/Sn/SiC with 3/8 ML and 6/8 BL coverages are chosen and shown in Supplementary Materials Figure S5(a,b) Both structures verified the high thermodynamic stabilities under preparation and application temperatures.

**Figure 3:**
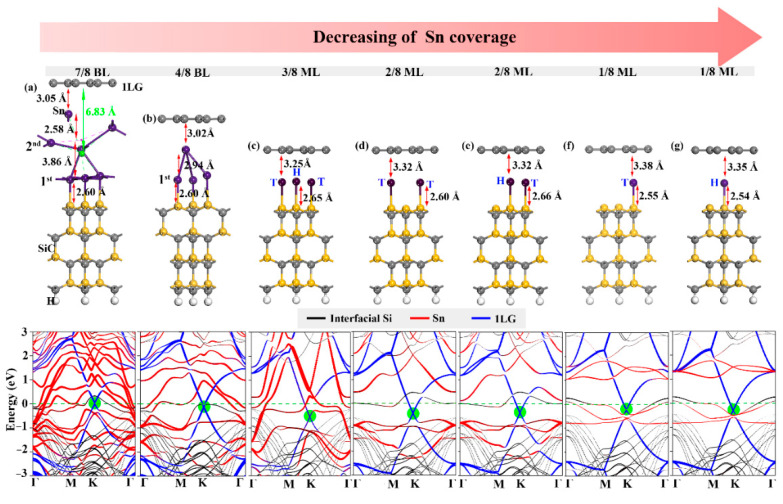
(**a**–**g**) Structures and band structures of 1LG/Sn/SiC with different Sn locations and coverages. The green ball in (**a**) represents the intercalated mass center of the Sn intercalation. In the band structures, the red, blue, and black lines represent the contribution of Sn intercalation, 1LG, and interfacial Si of SiC substrate, respectively. The green circles show the graphene Dirac point.

We also found the coverage-dependent regulation of electronic structure, but the effect and mechanism are different from Ge intercalation. Firstly, the distances between graphene and Sn intercalations are very large at high coverages. Normally, the total energy of the whole structure is contributed to two components—elastic contribution (positive effect) and electronic effect (negative effect) [50]. The intercalation structure has a significant influence on the structural relaxation under high coverage, resulting in the elastic contribution going beyond the electronic effect to be the dominant factor. Increasing the Sn coverage to 4/8 BL, the strong metallic character and sizable atomic radius of Sn atoms lead to great structural deformation (Figure 3). Therefore, the Sn layer is separated by a large distance at 4/8 BL coverage, making graphene closer to the Sn layer. Contrarily, the electronic effect becomes the dominant factor relative to the elastic contribution under low intercalation coverage (ML), and the graphene is slightly further away from the Sn intercalation.

**Figure 4:**
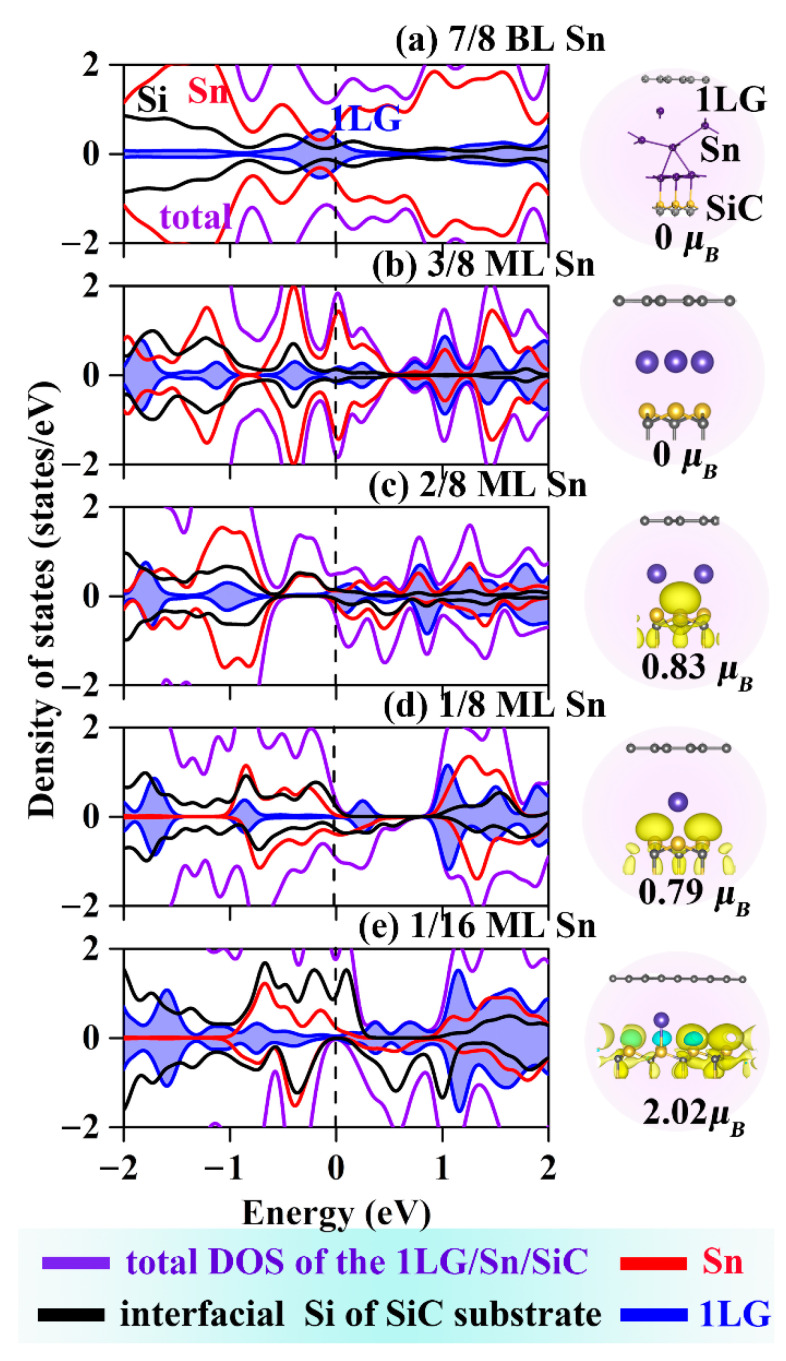
DOSs and spin densities for (**a**–**e**) 1LG/Sn/SiC with different coverages. Light blue and yellow isosurfaces represent positive and negative spin densities (±0.02 e/Å3), respectively. The purple lines represent the 1LG/Sn/SiC. The black, red, and blue lines correspond to the PDOS of interfacial Si of SiC substrate, Sn and 1LG, respectively.

On the other hand, the electron doping of graphene, owing to the Sn intercalation, is overall stronger than that for Ge intercalation. This difference can be attributed to the more metallic character of Sn than Ge, resulting in the more electron transfer to graphene. As a result, the Sn intercalation can be regulated to produce charge-neutral graphene experimentally [43]. Our calculations verified this conclusion for the 7/8 BL coverage, as shown in Figure 4a. Such a high coverage leads to the stabilization of the Sn adatom and huge deformation of the second Sn layer. We choose the mass center of the second Sn layer to measure the distance between graphene and the second Sn layer. It is found that the considerable deformation of the Sn layer increases the distance between graphene and the Sn layer to ~1 Å, which is larger than the distance between graphene and the Ge layer, and thus reduces the electron transfer with graphene and makes the graphene charge-neutral. Given the strong spin–orbit coupling effect of Sn with the coverage decreased to 1/8 ML, the band linearity around the Dirac point has been broken, as shown in Figure 4f,g, meaning that the electronic properties of graphene could be hardly improved relative to the freestanding graphene. Although this situation is reversed when the Sn coverage further decreases to 1/16 ML (Supplementary Materials Figure, the Sn intercalation with such a low coverage is energetically forbidden. Therefore, we suggest that an adequate Sn source is supplied in an experiment to obtain high coverage of Sn intercalation in order to modulate the charge-neutral freestanding graphene.

The spin DOS and the electron density of Sn-intercalated structures were calculated and showed in Figure 4. Due to Sn intercalations are analogously to the Ge intercalation, the effect of Sn intercalation coverage on the magnetic properties of the system is exactly the same as that of Ge intercalation. The maximum magnetic behavior of 1/16 ML Sn intercalated structures has magnetic moments of 2.02 μ_B_, which is the same as the maximum magnetic moment of Ge intercalated structure. Notably, when the Sn coverage reduces to 1/16 ML, the spin-up channel is metallic and the spin-down channel exhibits an electronic structure similar to the Dirac point of graphene. This change provides the capability to modulate the magnetism and ultra-fast electronic mobility in the same structure. The spin polarization ratio is defined as Equation (3).

(3) $$\delta =\left|\rho^{↑}{-\rho }^{↓}\right|{/(\rho }^{↑}{+\rho }^{↓})$$

where ρ^↑^ and ρ^↓^ are the absolute values of DOS at the Fermi level for spin-up and spin-down channels, respectively. For the 1/16 ML coverage, both the 1LG/Ge/SiC and 1LG/Sn/SiC is half-metals with 100% spin–polarization ratios at the Fermi level, which can be advantageous for nanoscale spintronic-electric applications.

According to our theoretical research results, experimental achievement of the p-type and charge-neutral graphene on SiC substrate is based on the precise regulation of Ge and Sn intercalation coverages. It should be noticed that the precise control of intercalation coverage has been achieved experimentally for many years. For example, Gierz et.al successfully traced the intercalation atom deposition process by using angle-resolved photoemission spectroscopy (ARPES) and precisely control the Bi intercalation coverages of 0→0.28→0.38→0.46 atoms/u.c. (the amount of Bi atoms per graphene unit cell) [25]. Moreover, more precise control of the atom deposition amount has been achieved for the thallium intercalation [47]. The controlling of the amount of thallium can span two orders of magnitude, and the minimum thallium concentration that can be controlled is 0.06% of the number of atoms in a graphene monolayer. It can be seen that the current experimental controlling precision of the intercalation layer coverage/concentration can completely achieve the concentration of Ge and Sn intercalations in our theoretical work. Therefore, it is possible to achieve bipolar regulation of graphene on SiC substrate by regulating Ge and Sn intercalation coverages experimentally.

### 2.3. Electronic Structure of Sn_1-x_Ge_x_ Intercalations

**Figure 5:**
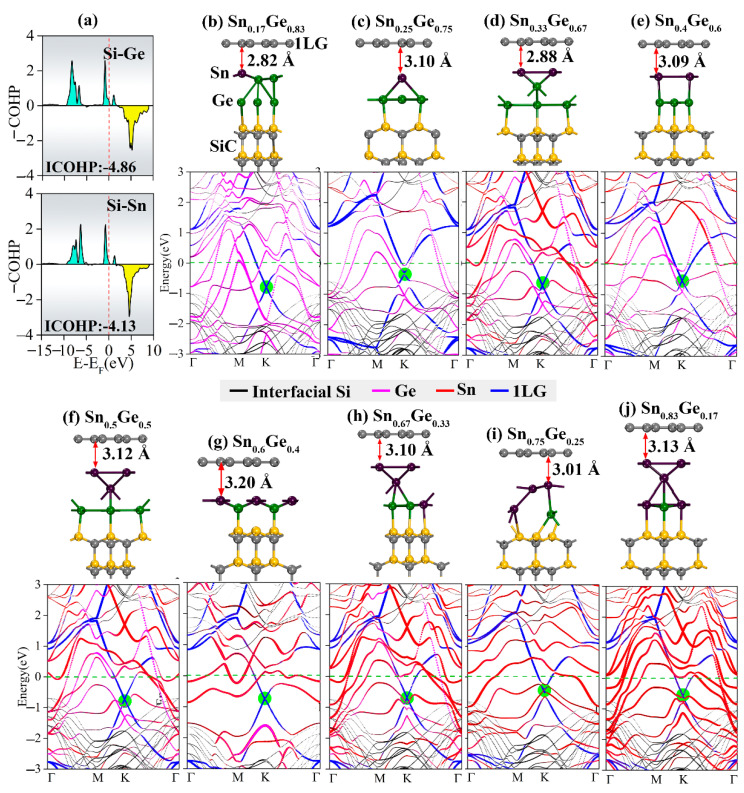
(**a**) ICOHP for the Ge or Sn interactions bonding with the Si atom in the SiC substrate. (**b**–**j**) Structures and band structures of Gr/Sn_1−x_Ge_x_/SiC with the Sn:Ge ratios of 1/5, 1/3, 2/4, 2/3, 3/3, 3/2, 4/2, 3/1, and 5/1, respectively. In the band structures, the red, pink, blue and black lines represent the contribution of Ge intercalation, 1LG, and interfacial Si of SiC substrate, respectively. The green circles show the graphene Dirac point.

According to an experimental report [46], Ge atoms could be deposited on the Sn atomic layer to form Sn_1-x_Ge_x_ alloy layers between graphene and the SiC substrate at 600–800 °C. The Sn:Ge ratio was modulated by changing the preparation temperature, and it was inferred that the doping characteristics of graphene could thus be affected. It was also experimentally found that the Ge atoms prefer to locate as the bottom layer and bind to the Si dangling bonds of the SiC substrate, while the Sn atoms are thus the top layer of the alloy intercalation. This phenomenon is confirmed by theoretical calculations. The calculated binding energy between Ge and interfacial Si atoms is −4.19 eV, while that of Sn is −3.81 eV, indicating that Ge atoms are more preferable to bind with the Si-terminated surface than Sn. The calculated ICOHP values of Ge-Si and Sn-Si are −4.86 and −4.13 respectively (Figure 5a), further confirming that Si more favorably combines with Ge than Sn. Therefore, models of Sn_1-x_Ge_x_ intercalations were constructed by placing the Ge atomic layer at the bottom to bind with Si, and then, an Sn atomic layer is placed between the Ge layer and graphene.

The optimized 1LG/Sn_1-x_Ge_x_/SiC configurations and the corresponding band structures are shown in Figure 5. Similarly, there is only a weak van der Waals interaction between graphene and Sn1-xGex intercalations—the distances between them with different Sn:Ge ratios remain around 3.0 Å, which is ~0.2–0.4 Å smaller than those of 1LG/Ge/SiC and 1LG/Sn/SiC. This observation indicates a more substantial electron transfer from Sn_1-x_Ge_x_ to graphene, leading to the doping of graphene by more electrons. More importantly, because Sn and Ge are metallic, the Sn_1-x_Ge_x_ intercalation layers have a more substantial metallic effect than the isolated Ge or Sn layers, leading to more substantial electron transfer to graphene. This phenomenon could be reflected by the strong n-type behavior of graphene, which was not reversed to p-type by modulating the Sn:Ge ratio. When the Sn:Ge ratio was small, the linear dispersion of the graphene Dirac point remains in good standing. However, as the Sn:Ge ratio increases, the contribution of Ge atoms in the conduction band smoothly changes to that of Sn, and the linear dispersion of the graphene band deteriorates owing to the complicated hybridization of the electronic states of Ge, Sn, and interfacial Si. It is therefore hard to obtain charge-neutral and p-type graphene by Sn_1-x_Ge_x_ alloy intercalation.

## 3. Materials and Methods

**Figure 6:**
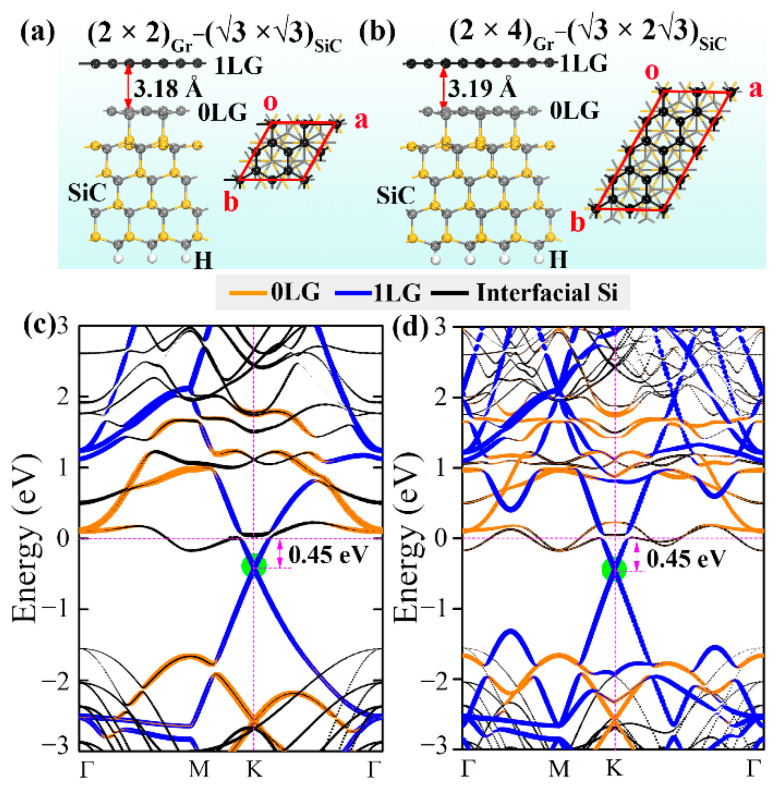
(**a**,**b**) (2 × 2)_Gr_ − (√3 × √3)_SiC_ and (2 × 4)_Gr_ − (√3 × 2√3)_SiC_ graphene−SiC interface atomic configurations, respectively. Black, gray, and yellow balls represent C atoms in graphene, C atoms in SiC, and Si atoms, respectively. (**c**,**d**) The corresponding electronic band structures. The orange, blue, and black lines represent the contribution of 0LG, 1LG, and interfacial Si of SiC substrate, respectively. The green circles show the location of the graphene Dirac point.

First-principles calculations were performed based on the Vienna ab initio simulation package (VASP) [51,52] in conjunction with projector augmented wave (PAW) [53]. The electrons in C 2s^2^2p^2^, Si 3s^2^3p^2^, Ge 4s^2^4p^2^, and Sn 5s^2^5p^2^ were treated as valence electrons. Generalized gradient approximation (GGA) in the Perdew–Burke–Ernzertaly (PBE) form [54] was adopted for the exchange–correlation interaction to optimize the configurations and describe the electronic properties of all the investigated structures. A 450 eV cutoff energy was used for the plane wave basis, and the convergence criteria were chosen as 0.05 eV/Å and 10^−4^ eV/atom for the residual force and total energy, respectively. To provide a good approximation of the experimentally reported (6√3 × 6√3) R30° reconstruction [9], an interface model of graphene and SiC substrate was constructed by placing a 2 × 2 graphene sheet (8 C atoms) on top of a (√3 × √3) R30° 6H-SiC(0001) surface ((2 × 2)Gr − (√3 × √3)SiC supercell) that contains four SiC layers with H saturating the C bonds located at the bottom, as shown in Figure 6a. This model corresponds to a 4.1%-stretched graphene cell and a 4.4%-compressed SiC substrate. An 18 Å thick vacuum layer was adopted perpendicular to the interface to prevent artificial interactions between the adjacent supercells. Normally, the buffer carbon layer was abbreviated as 0LG, and the first layer of the quasi-freestanding graphene was abbreviated as 1LG. Detailed information of the intercalated system configurations (Supplementary Materials Figure S1) was included in the Supplementary Materials. To clarify the influence of the intercalations on the electronic structure of graphene, “fat band” technology was used to observe the occupation of various elements in the band structures. In addition, to further explore the effect of intercalation coverage (correspond to different amount of atomic vacancies) on the electronic structure of graphene, we doubled the size of the (2 × 2)Gr − (√3 × √3)SiC supercell along the y axis to obtain a (2 × 4)Gr − (√3 × 2√3)SiC supercell, as shown in Figure 6b. A 7 × 7 × 1 Monkhorst-Pack k-mesh was used for structural relaxation and electronic structure calculations. Long-range van der Waals interaction (essential for describing the graphene-intercalation interactions) was included by Grimme’s semi-empirical correction (DFT-D2) [55]. The ab initio molecular dynamics (AIMD) simulations were performed at 300, 900, and 1200 K using a canonical ensemble (NVT) within each time step of 1 fs to evaluate the thermodynamic stability of the intercalated structures.

## 4. Conclusions

In summary, we investigated the effect of group-IV Ge, Sn, and Sn_1-x_Ge_x_ metal intercalations on the structure and electronic structure of epitaxial graphene on SiC substrate by performing DFT calculations. It is found that the buffer carbon layer could turn into monolayer quasi-free-standing graphene by intercalating these metal layers between graphene and SiC substrate, and the doping properties of graphene depend on the type of metal intercalations and the intercalation coverages. The Ge intercalation induces graphene ambipolar doping, and the p-type graphene could be obtained with 4/8 and 7/8 BL coverages. The p-type graphene originates from the strong interaction between the adatom Ge and its nearby Ge layer. On the other hand, the charge-neutral graphene is tailored by the Sn intercalation with as high coverage as 7/8 BL since the charge transfer between graphene and the Sn layer can be eliminated by the massive deformation of the Sn layer and the resulting enlarged distance between them. Further studies show that the group-IV metal intercalations induce interesting magnetic properties to the entire structure while keeping graphene free of magnetism. These results help understand the effect and mechanism of main-group metal intercalation on the electronic and magnetic property regulation of graphene. It also provides a new path to develop novel spintronic-electric devices based on these composite layered structures.

## Figures and Tables

**Table 1:** Formation energies (*E*_f_ in eV per atom) and intercalation energies (*E*_I_ in eV) for different Ge coverages.

Ge Coverage	7/8 BL	6/8 BL	5/8 BL	4/8 BL	3/8 ML	2/8 ML	2/8 ML	1/8 ML	1/8 ML	1/16 ML	1/16 ML
Ge location	-	-	-	-	-	T + T	T + H	T	H	T	H
*E*_f_	0.42	0.18	0.26	0.29	0.68	0.20	0.19	−1.68	−1.62	−3.38	−3.33
*E*_I_	−3.01	−1.10	−1.29	−1.15	−2.03	−0.40	−0.38	1.68	1.62	3.38	3.33

**Table 2:** Bond lengths (Å) and ICOHP values of Ge-Ge bonds in 1LG/Ge/SiC with a 7/8 BL coverage.

Bond	Length (Å)	ICOHP
Ge_1_—-Ge_2_	2.47	−3.74
Ge_1_—Ge_3_	2.72	−2.48
Ge_1_—Ge_4_	2.47	−3.74
Ge_2_—Ge_3_	3.18	−0.49
Ge_3_—Ge_4_	3.38	−0.49
Ge_2_—Ge_4_	3.59	−0.08

**Table 3:** Formation energies (*E*_f_ in eV per atom) and intercalation energies (*E*_I_ in eV) for different Sn coverages.

Sn Coverage	7/8 BL	6/8 BL	5/8 BL	4/8 BL	3/8 ML	2/8 ML	2/8 ML	1/8 ML	1/8 ML	1/16 ML	1/16 ML
Sn location	-	-	-	-	-	T + T	T + H	T	H	T	H
*E*_f_	0.28	0.04	0.06	0.12	0.66	0.66	0.62	0.27	0.27	−4.38	−4.35
*E*_I_	−1.92	−0.21	−0.30	−0.48	−1.98	−1.32	−1.24	−0.27	−0.27	4.38	4.35

## Data Availability

Not applicable.

## Supplementary Materials

The following supporting information can be downloaded at: https://www.mdpi.com/article/10.3390/molecules27249004/s1, Figure S1: Configurations of Ge and Sn intercalation systems with different coverages; Figure S2 Distribution of the electron localization function (ELF) in the (11(_)0) plane of (a) 0LG/SiC, (b) 1LG/Ge/SiC, and (c) 1LG/Sn/SiC with 3/8 ML coverage. The black dotted boxes were used to frame the interface between graphene and the substrate or intercalation; Figure S3: AIMD simulations of (a) 1LG/Ge/SiC with 3/8 ML coverage and (b) 1LG/Ge/SiC with 6/8 BL coverage at 300 K, 900 K and 1200 K; Figure S4: (a-d) Structures and electronic band structures corresponding to the atomic structures of 1LG/Ge/SiC with different Ge locations and coverages. In the band structures, the pink, blue and black lines represent the contribution of Ge intercalation, 1LG, and interfacial Si of SiC substrate, respectively. The green circles show the location of the graphene Dirac point; Figure S5: AIMD simulations of (a) 1LG/Sn/SiC with 6/8 BL coverage and (b) 1LG/Sn/SiC with 6/8 BL at 300 K, 900 K and 1200 K; Figure S6: (a-d) Structures and electronic band structures corresponding to the atomic structures of 1LG/Sn/SiC with different Sn locations and coverages. In the band structures, the red, blue and black lines represent the contribution of Ge intercalation, 1LG, and interfacial Si of SiC substrate, respectively. The green circles show the location of the graphene Dirac point; Table S1: Ge/Sn coverage-dependent Fermi level (E_f_ in eV), doping type of graphene, and magnetic moment (μ_B_) of the intercalation structures. The numbers in brackets represent the magnetic moment of 1LG. References [56,57] are cited there.
