# Supplementary material for: Regulating the Electronic Structure of Freestanding Graphene on SiC by Ge/Sn Intercalation: A Theoretical Study
Source: Molecules. 2022 Dec 17;27(24):9004. doi: 10.3390/molecules27249004 (PMC9788586; doi:10.3390/molecules27249004)
Supplement: Supplementary file 1 [file molecules-27-09004-s001.zip › molecules-2049128-supplementary.pdf]

Supplementary Information

Regulating the electronic structure of freestanding graphene on SiC by Ge /Sn intercalation: A theoretical study

Xingyun Luo[1], Guojun Liang[1], Yanlu Li[1]*, Fapeng Yu[1], Xian Zhao[2]*

[1]State Key Lab of Crystal Materials, Institute of Crystal Materials, Shandong University, Jinan 250100, China

[2]Center for Optics Research and Engineering of Shandong University, Shandong University, Qingdao 266237, China

Corresponding Authors

Y. Li, *Email: liyanlu@sdu.edu.cn

X. Zhao, *Email: xianzhao@sdu.edu.cn

1. Constructed intercalation system

According to the experiments, the Ge, Sn and Sn_xGe_{1-x} layers were intercalated between the 0LG and SiC substrate to turn the 0LG into 1LG [1-3]. The other intercalation locations, such as chemisorbing on graphene, substituting a Si atom at the SiC surface, etc., seriously reduce the linear dispersion of graphene [4]. Therefore, we only investigate the case of intercalations between graphene and SiC substrate. Because of the lack of sufficient experimental evidence for an ordered reconstruction of these intercalation structures, the Ge/Sn intercalation layers were constructed by saturating the Si dangling bonds on the SiC substrate. The Sn_xGe_{1-x} alloy intercalation layers were obtained by replacing the interfacial Si or C with Ge or Sn. Meanwhile, the experimental reports implied that the doping properties of graphene are determined by the thickness of Ge intercalation [1]. Therefore, we consider the Ge, Sn and their alloy layers with different number of layers and with different concentration of atomic vacancies, described by the coverage of intercalations for simplicity. For example, for monolayer (ML) intercalation, the Ge/Sn atoms occupy two positions relative to graphene: one below the C atom of graphene, labeled as position T; the other below the center of the hexagonal lattice of graphene, labeled as position H. The coverage in such intercalation layer was calculated based on 8 or 16 C atoms in a (2 × 2) or (2 × 4) ML graphene structure, respectively. In the $(2 \times 2)_{Gr}$-$(\sqrt{3} \times \sqrt{3})_{SiC}$ configuration, there are three Ge/Sn atoms: two at position T and one at position H. Leaving one Ge/Sn atom represents a coverage of 1/8 ML, and two Ge/Sn atoms represent a coverage of 2/8 ML. For the bilayer (BL) intercalations, seven Ge/Sn represent a coverage of 7/8 BL. Similarly, in the $(2 \times 4)_{Gr}$-$(\sqrt{3} \times 2\sqrt{3})_{SiC}$ configuration, one Ge/Sn atom represents a coverage of 1/16 ML.

2. Formation energy and intercalation energy

The formation energies [5] based on the configurations in Figure S1 in the Supporting Information were calculated to reflect the possible and preferable coverage ranges of the intercalations in terms of energy. It is defined as

$$E_{\mathrm{f}} = [(n\mu + E_{ini}) - E_{total}]/n$$

where n and μ are the number and chemical potential of intercalation atoms, E_{ini} and E_{total} denote the energies of the structures without and with intercalations, respectively. A positive value of E_f indicates the system energetically favorable.

The intercalation energy (E_I) is used to evaluate the structural stability of the intercalation structures. It can be calculated according to [6]

$$E_I = E(graphene/Ge(Sn)/SiC) - E(graphene/SiC) - E(Ge(Sn))$$

where $E(graphene/Ge(Sn)/SiC)$, $E(graphene/SiC)$, and $E(Ge(Sn))$ are the energies of the graphene/Ge(Sn)/SiC, the graphene/SiC, and the Ge (Sn) atom in their bulk states, respectively. Here, a negative value indicates that the intercalation is thermodynamically favorable.

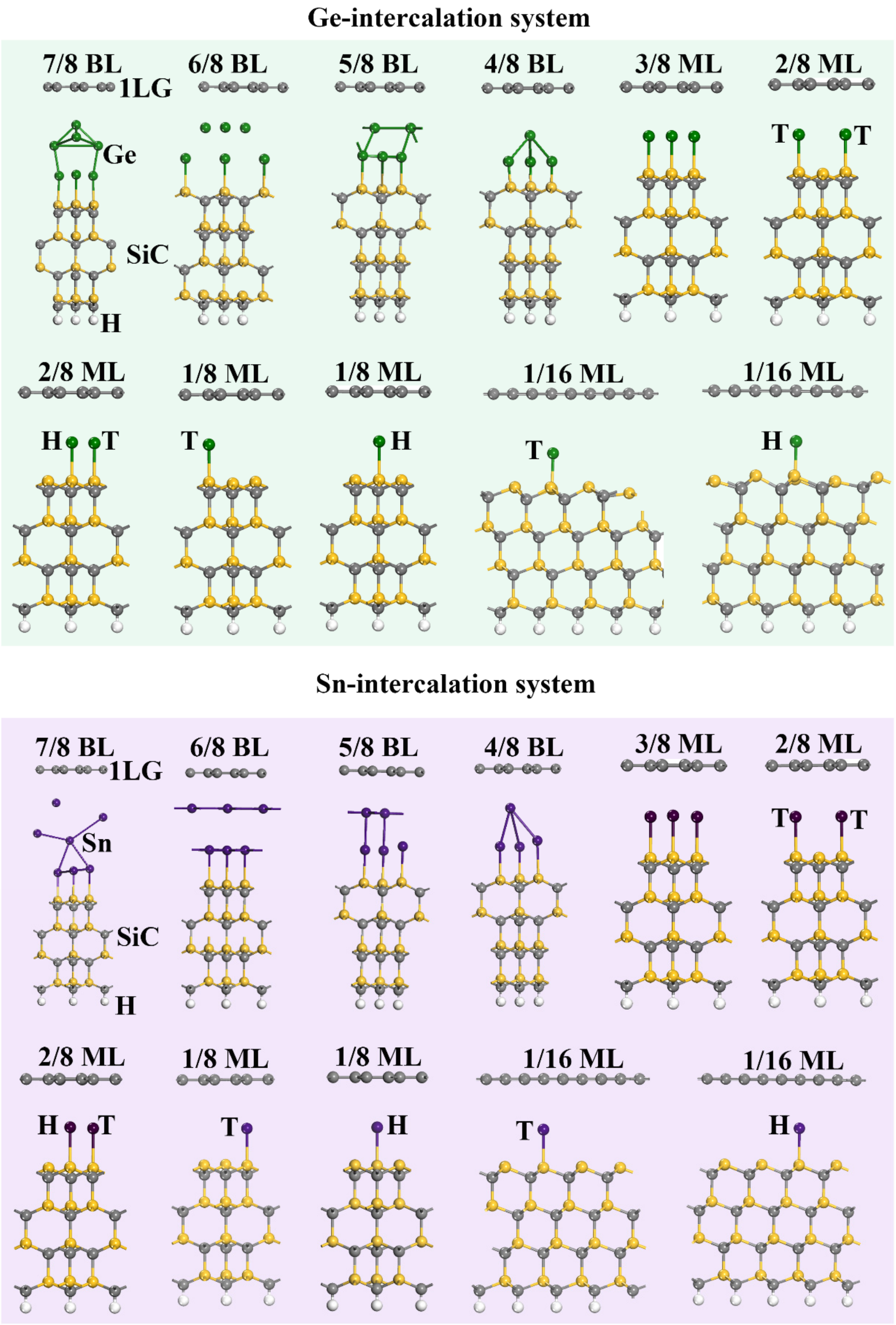

Fig. S1. Configurations of Ge and Sn intercalation systems with different coverages.

3. The decoupling of Ge/Sn intercalation

A lot of experimental literatures showed that the 1LG was prepared by decoupling the 0LG from the SiC substrate via intercalations [7-11]. Therefore, we investigate the effect of intercalations on decoupling the 0LG and regulating the electronic structure of 1LG. First, we compared the electron localization function (ELF) of structures without intercalation and with a coverage of 3/8 ML Ge and Sn intercalations to explore the chemical bonding nature of graphene, intercalation layer, and substrate in Figure S2. The red regions in ELF normally correspond to high values of the electron localization function and thus show the chemical bonding interaction. We can clearly see the strong electronic localization at the 0LG/SiC interface without intercalation (framed by the black dotted box in Figure S2a), while there is a clear blue gap between the electron distribution of 1LG and the remaining structure after both Ge and Sn intercalations (Figures. S3b and S3c). This indicates that electron distribution of intercalations has not been extended to graphene, and the strong electron localization between graphene layer and the remaining structures has been destroyed. On the contrary, the intercalations show obvious chemical interaction with SiC substrate. This verifies that the Ge and Sn intercalations can completely decouple the interaction between the 0LG and SiC substrate, making the 0LG become freestanding 1LG. This is consistent with previous reports [4] that the Ge layer between graphene and substrate can detach the buffer layer from the substrate. It is noted that the intercalations completely saturate the Si dangling bonds by forming strong Ge/Sn-Si covalent bonds, whereas maintain a Ge/Sn dangling bond perpendicular to the graphene rather than form Ge/Sn-C bonds. This observation

is sufficient to illustrate that the energy loss in the sp^2 planarity of the graphene is higher than the energy gain from forming the covalent Ge/Sn-C bonds.

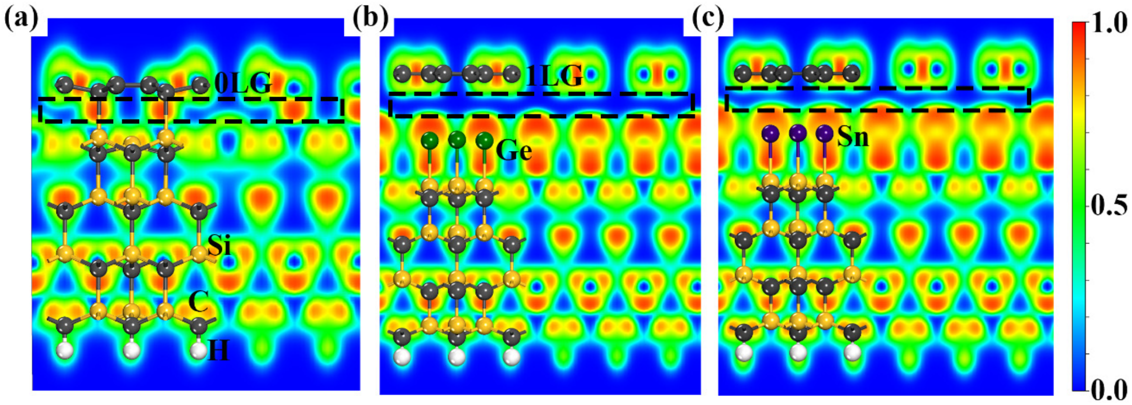

Figure S2. Distribution of the electron localization function (ELF) in the $(1\bar{1}0)$ plane of (a) 0LG/SiC, (b) 1LG/Ge/SiC, and (c) 1LG/Sn/SiC with 3/8 ML coverage. The black dotted boxes were used to frame the interface between graphene and the substrate or intercalation.

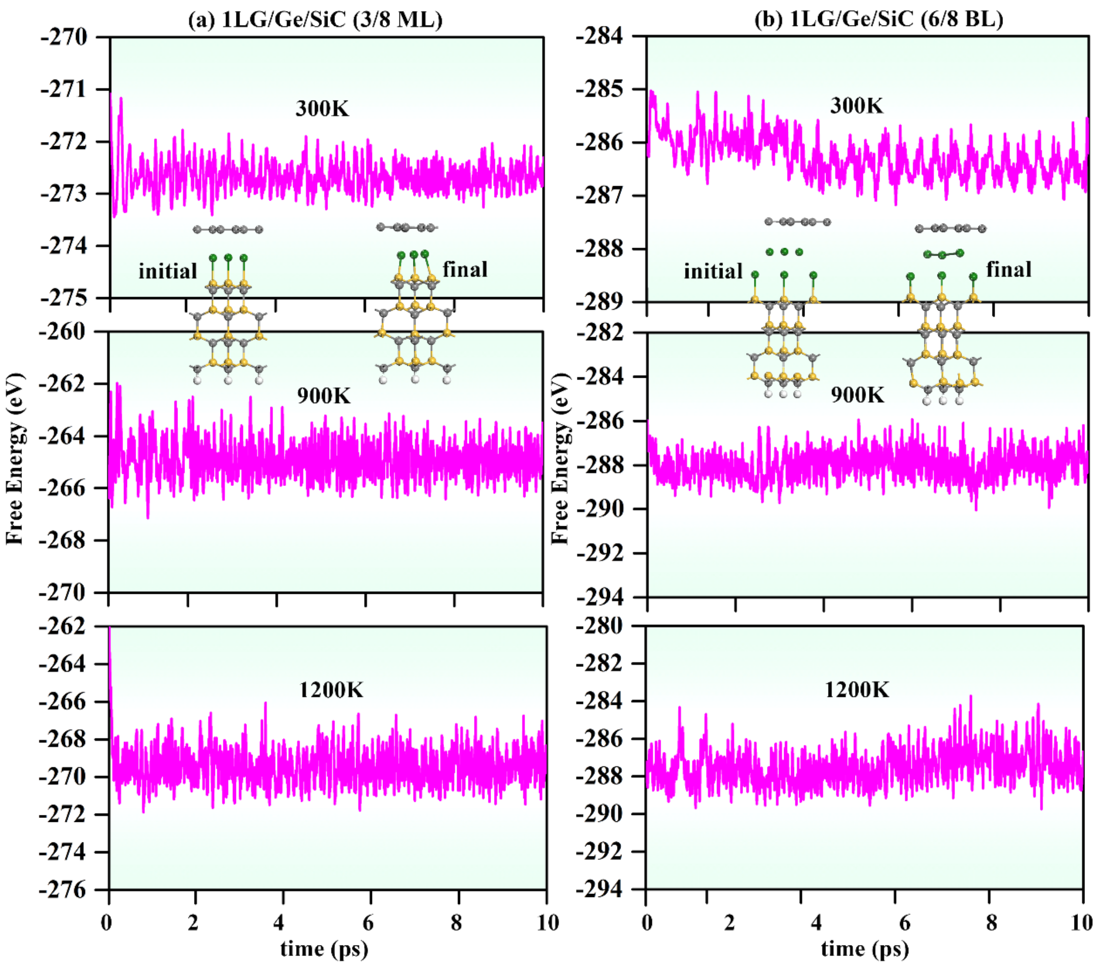

Fig. S3. AIMD simulations of (a) 1LG/Ge/SiC with 3/8 ML coverage and (b) 1LG/Ge/SiC with 6/8 BL coverage at 300 K, 900 K and 1200 K.

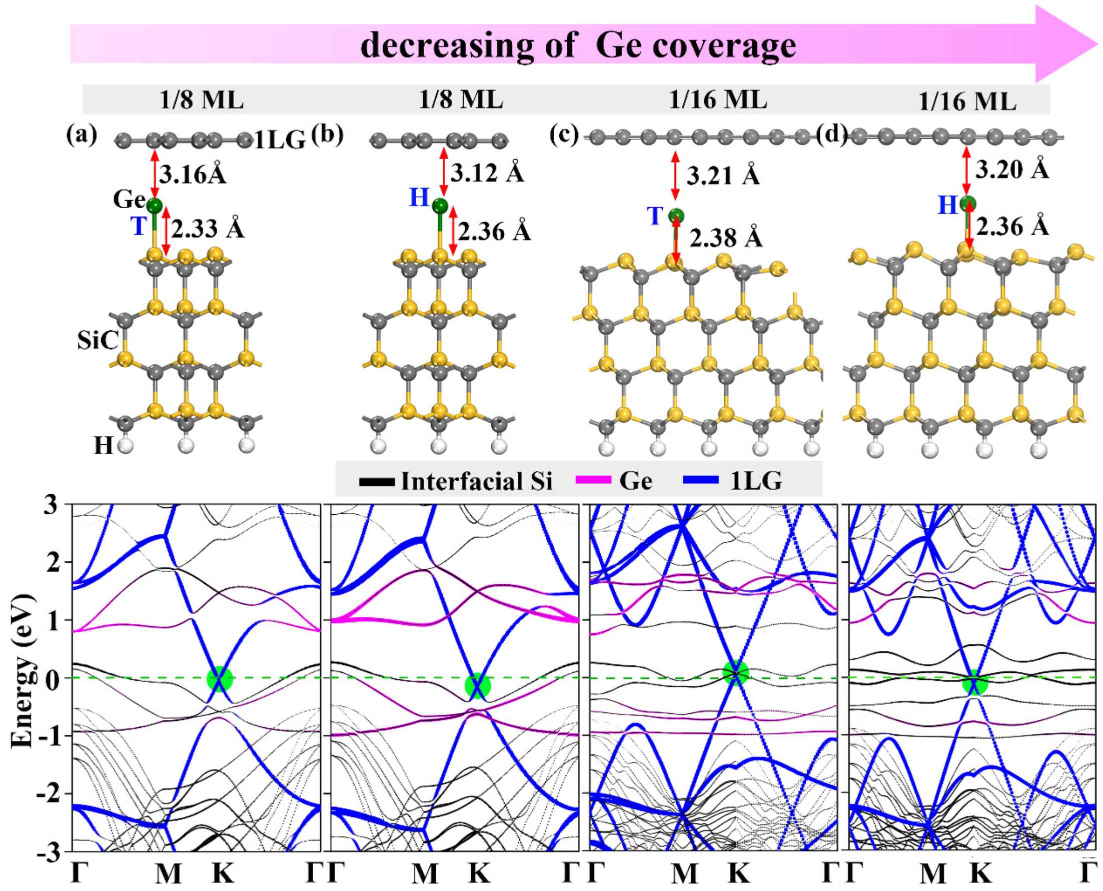

Fig. S4. (a-d) Structures and electronic band structures corresponding to the atomic structures of 1LG/Ge/SiC with different Ge locations and coverages. In the band structures, the pink, blue and black lines represent the contribution of Ge intercalation, 1LG, and interfacial Si of SiC substrate, respectively. The green circles show the location of the graphene Dirac point.

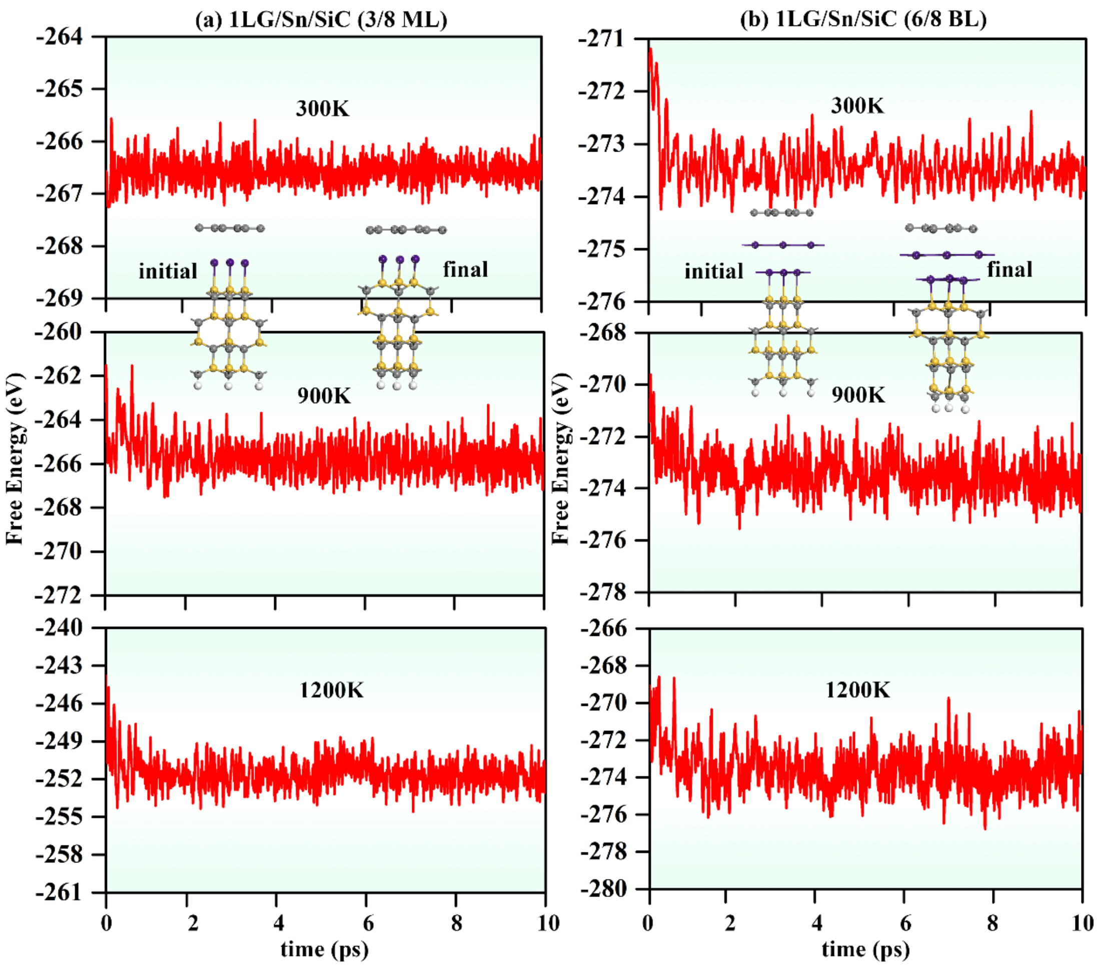

Fig. S5. AIMD simulations of (a) 1LG/Sn/SiC with 6/8 BL coverage and (b) 1LG/Sn/SiC with 6/8 BL at 300 K, 900 K and 1200 K.

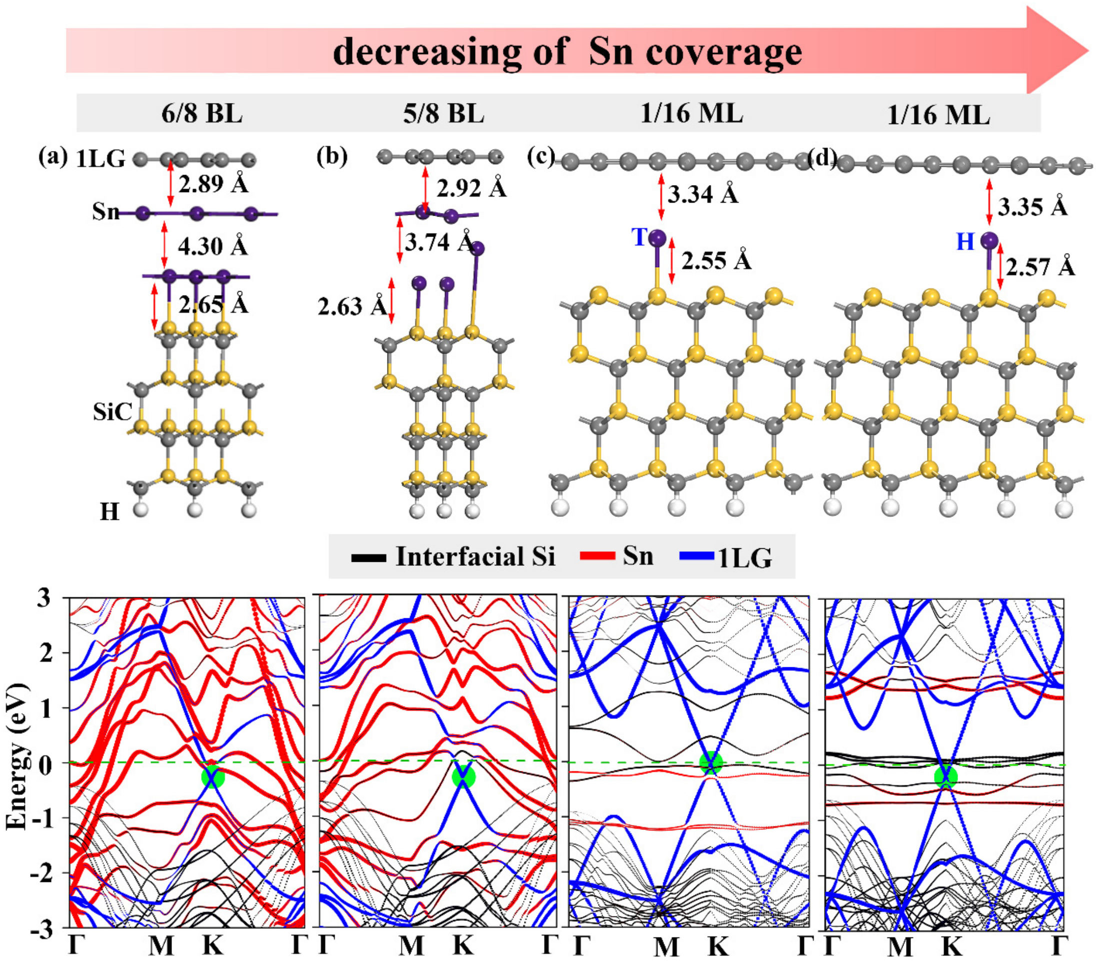

Fig. S6. (a-d) Structures and electronic band structures corresponding to the atomic structures of 1LG/Sn/SiC with different Sn locations and coverages. In the band structures, the red, blue and black lines represent the contribution of Ge intercalation, 1LG, and interfacial Si of SiC substrate, respectively. The green circles show the location of the graphene Dirac point.

Table S1. Ge/Sn coverage-dependent Fermi level (ε_f in eV), doping type of graphene, and magnetic moment (μ_B) of the intercalation structures. The numbers in brackets represent the magnetic moment of 1LG.

Coverage	Location	Ge intercalation			Sn intercalation		
		ε_f	Doping type	μ_B	ε_f	Doping type	μ_B
7/8 BL	/	2.38	*p*	0 (0)	2.09	neutral	0 (0)
6/8 BL	/	2.59	*n*	0 (0)	2.35	*n*	0 (0)
5/8 BL	/	2.17	*n*	0 (0)	2.11	*n*	0 (0)
4/8 BL	/	1.71	*p*	0 (0)	1.74	*n*	0 (0)
3/8 ML	/	1.20	*n*	0 (0)	1.60	*n*	0 (0)
2/8 ML	T+T	1.42	*n*	0.35 (0)	1.48	*n*	0.83 (0)
2/8 ML	T+H	1.37	neutral	0.36 (0)	1.46	*n*	0.83 (0)
1/8 ML	T	0.98	neutral	0.64 (0)	1.30	*n*	0.79 (0)
1/8 ML	H	1.00	*n*	0.67 (0)	1.26	*n*	0.78 (0)
1/16 ML	T	1.07	*p*	1.92 (0)	1.04	neutral	2.02 (0)
1/16 ML	H	1.18	neutral	1.99 (0)	1.09	*n*	1.91 (0)

4. References

1 Kim, H.; Dugerjav, O.; Lkhagvasuren, A.; Seo, J.M. Origin of ambipolar graphene doping induced by the ordered Ge film intercalated on SiC(0001). *Carbon* **2016**, *108*, 154-164.

2 Kim, H.; Dugerjav, O.; Lkhagvasuren, A.; Seo, J.M. Charge neutrality of quasi-free-standing monolayer graphene induced by the intercalated Sn layer. *J. Phys. D Appl. Phys.* **2016**, *49*, 135307.

3 Kim, H.; Dugerjav, O.; Lkhagvasuren, A.; Seo, J.M. Doping modulation of quasi-free-standing monolayer graphene formed on SiC(0001) through $Sn_{1-x}Ge_x$ intercalation. *Carbon* **2019**, *44*, 549-556.

4 Deretzis, I.; La Magna, A. Ab initio study of Ge in epitaxial graphene on SiC(0001). *Appl. Phys. Express* **2011**, *4*, 125101.

5 Wang, J.; Kim, M.; Chen, L.; Ho, K.; Tringides, M.; Wang, C.; Wang, S. Manipulation of electronic property of epitaxial graphene on SiC substrate by Pb intercalation. *Phys. Rev. B* **2021**, *103*, 085403.

6 Kotsakidis, J.C.; Grubišić-Čabo, A.; Yin, Y.; Tadich, A.; Myers-Ward, R.L.; Dejarld, M.; Pavunny, S.P.; Currie, M.; Daniels, K.M.; Liu, C.; Edmonds, M.T.; Medhekar, N.V.; Gaskill, D.K.; Vázquez de Parga, A.L.; Futrer, M.S. Freestanding *n*-doped graphene via intercalation of calcium and magnesium into the buffer layer-SiC(0001) interface. *Chem. Mater.* **2020**, *32*, 6464-6482.

7 Guisinger, N.P.; Rutter, G.M.; Crain, J.N.; First, P.N.; Stroscio, J.A. Exposure of epitaxial graphene on SiC(0001) to atomic hydrogen. *Nano Lett.* **2009**, *9*, 1462-1466.

8 Tanabe, S.; Talamura, M.; Harada, Y.; Kageshima, H.; Hibino, H. Effects of hydrogen intercalation on transport properties of quasi-free-standing monolayer graphene. *Jpn. J. Appl. Phys.* **2014**, *53*, 04EN01.

9 Bom, N.M.; Oliveira Jr., M.H.; Soares, G.V.; Radtke, C.; Lopes, J.M.J.; Riechert, H. Synergistic effect of H_2O and O_2 on the decoupling of epitaxial monolayer graphene from SiC(0001) via thermal treatments. *Carbon* **2014**, *78*, 298-304.

10 Gao, T.; Gao, Y.; Chang, C.; Chen, Y.; Liu, M.; Xie, S.; He, K.; Ma, X.; Zhang, Y.; Liu, Z. Atomic-scale morphology and electronic structure of manganese atomic layers underneath epitaxial graphene on SiC(0001). *ACS Nano* **2012**, *6*, 6562.

11 Li, Y.; West, D.; Huang, H.; Li, J.; Zhang, S.B.; Duan, W. Theory of the Dirac half metal and quantum anomalous Hall effect in Mn-intercalated epitaxial graphene. *Phys. Rev. B* **2015**, *92*, 201403(R).
